# The association between climate-smart agriculture practices adoption and farm income and wealth of small-scale urban crop farmers in eThekwini municipality, with implications for food and nutrition security

**DOI:** 10.3389/fnut.2026.1792895

**Published:** 2026-05-21

**Authors:** Nolwazi Z. Khumalo, Melusi Sibanda, Lelethu Mdoda

**Affiliations:** 1 Department of Agriculture, University of Zululand, Kwadlangezwa, South Africa; 2 Discipline of Agricultural Economics, School of Agriculture, Earth and Environmental Sciences, University of KwaZulu-Natal, Pietermaritzburg, South Africa

**Keywords:** climate change adaptation, farm productivity, income, South Africa, urban agriculture, wealth

## Abstract

**Introduction:**

South Africa is particularly vulnerable to the effects of climate change due to its reliance on climate-sensitive livelihoods and limited adaptive capacity. Climate-smart agriculture (CSA) emerges as a key adaptation strategy to climate change, while enhancing agricultural productivity and improving income security with direct or indirect implications for food and nutrition security. Therefore, this study assessed the association between CSA practices used by small-scale urban crop (SSUC) farmers and farm income and wealth in the eThekwini (ETH) Municipality.

**Methods:**

The CSA adoption relied on a composite index computed from various practices and their intensities, including agroforestry (A), conservation agriculture (CA), crop diversification (CD), crop rotation (CR), cover crop (CC) use, drought-tolerant (DT) crops, mulching (M), organic manure (OM) use, wetland (W) use, and soil conservation (SC). The study collected data from 412 SSUC farmers selected through a multi-stage sampling procedure, with purposive selection and snowball sampling applied at the final stage. The study utilised descriptive analysis and conditional mixed processes (CMP) to analyse data.

**Results:**

Descriptive results underscore constraints imposed by demographic and institutional factors on SSUC farmers. The CMP model demonstrates a significant and positive association between CSA practices and the wealth index and farm income, jointly modelling adoption and welfare outcomes while accounting for potential selection bias. The CSA adoption showed a positive association with the wealth index and farm income. However, the estimated association between CSA practices adoption and income are relatively modest. They should be noted with caution, as substantial income gains and asset accumulation may become apparent in the long term, along with enhanced resilience to climate variability. Several socio-economic characteristics also showed significant associations, some with counterintuitive associations, for example, agricultural training, off-farm income, and hired labour, which warrant further exploration of the UA dynamics.

**Conclusion:**

The paper underscores the need to improve access to context-specific and outcome-oriented agricultural training and extension services, provided that existing training shows a weak association with income-enhancing CSA implementation, alongside strengthening economically productive farmer groups and continued research into CSA adoption to enhance farm income and wealth outcomes among small-scale urban farming households.

## 1 Introduction

Climate change is one of the most pressing global issues of the 21st century, with rising global temperatures and shifting precipitation patterns (1, 2). Sub-Saharan Africa (SSA) will likely experience above-average temperature increases and reduced rainfall, exacerbating extreme weather events such as droughts and floods (3, 4). These climatic shifts pose severe risks to developing nations, where economies rely on climate-sensitive livelihoods and have limited capacity for adaptation (5). Climate change negatively impacts food production, leading to declining agricultural productivity, livestock losses, food price fluctuations, and disruptions to market supply (6). Small-scale farmers in SSA, who are heavily reliant on rain-fed agriculture, face increasing vulnerability, thereby heightening the risk of poverty (7). Urbanisation and population growth further limit income security, especially for small-scale urban crop (SSUC) farmers (8). Given these challenges, addressing agricultural adaptation is imperative for achieving the Sustainable Development Goals (SDGs), particularly SDG 1 (No Poverty), SDG 2 (Zero Hunger), and SDG 13 (Climate Action). In this paper, SSUC farmers refer to individuals or farming households practising crop production within the urban confines of the eThekwini (ETH) Municipality. The SSUC farmers primarily farm on small plots of land less than 2 hectares (9), relying on family labour, and produce mainly for their own consumption and surplus for economic gain. Small-scale urban farmers typically operate with limited land, low capital, and a lack of formal agricultural support (10).

Urbanisation is rapidly transforming food systems globally, with the urban population rising from 30% in 1950 to 57% in 2021 and projected to reach 68% by 2050 (11). While urbanisation is synonymous with economic growth, in many developing countries, it leads to unemployment, poor living conditions, and food insecurity (12). Climate change and environmental degradation further drive rural-to-urban migration, increasing pressure on urban food supply and demand (13, 14). South Africa, one of the most urbanised countries in Africa, has 67% of its population living in cities, with this percentage expected to rise to 80% by 2050 (15). Despite economic opportunities, rapid urbanisation exacerbates poverty, land degradation, and unemployment, affecting food access, particularly in low-income communities (16, 17). In KwaZulu-Natal (KZN) Province, particularly in the eThekwini (ETH) Municipality, climate change has led to increased rainfall variability, frequent and intense droughts, rising temperatures, and extreme weather events such as flooding (18). Recurrent droughts constrain water availability for crop production, and flooding causes crop damage, particularly in low-lying and informal farming areas. The ETH Municipality, a coastal area, is vulnerable to heat stress and prone to storms and flooding, which disrupt agricultural activities, damage infrastructure, and exacerbate production risks for SSUC farmers. These climate stressors directly threaten urban crop production, food security and household income. Many urban households depend on income to purchase food, yet disparities in food access contribute to poor dietary choices and diet-related health issues (19, 20). In response, South African municipalities, such as Cape Town and Johannesburg, have introduced urban agriculture (UA) policies to improve food security (21, 22).

Urban agriculture plays a crucial role in providing livelihood opportunities and food security for SSUC farmers, particularly low-income households. It aligns with global and national strategies to achieve SDGs, including SDG 3 (good health and well-being), SDG 10 (reduced inequalities), and SDG 11 (sustainable cities and communities) (23). Urban agriculture supports climate resilience by integrating sustainable techniques such as hydroponics and aquaponics, which optimise yields while conserving resources (24). Furthermore, local food production reduces reliance on long supply chains, mitigates food price volatility, and increases urban resilience to climate shocks (25, 26). Given its potential for sustainable food production, UA is gaining traction as a climate-smart strategy that can enhance both food and income security (27).

Implementing UA with climate-smart agriculture (CSA) practices enhances sustainability and economic resilience in the face of climate change. Climate-smart agriculture, as used in this paper, refers to the use of agronomic practices that enhance farm productivity and resilience to climate variability while supporting sustainable resource use in urban farming systems. Various on-farm CSA practices documented in the literature and commonly accessible by SSUC farmers include crop diversification (CD), crop rotation (CR), mulching (M), use of drought-tolerant (DT) crop varieties, organic manure (OM) application, soil conservation (SC), agroforestry (A), conservation agriculture (CA), cover cropping (CC), and wetland (W) use (28). CSA aims to increase agricultural productivity, reduce greenhouse gas emissions, and improve farmers’ adaptation to climate change (29). Climate-smart agriculture’s triple-win objective fosters synergies among locally adapted strategies to strengthen resilience and reduce environmental footprints (30). Despite these benefits, CSA adoption remains low in South Africa, particularly among SSUC farmers who face financial, social, and institutional barriers (31–33). In the ETH Municipality, where UA significantly contributes to household livelihoods, it presents a relevant case for examining the adoption of CSA and its economic implications. Studies suggest that adopting CSA practices can improve farm productivity, mitigate climate risks, and enhance income, supporting sustainable agricultural development and agribusiness (34, 35). However, the economic benefits of CSA adoption in urban farming contexts remain underexplored (36). The underexploitation of CSA’s economic benefits in urban contexts reflects a combination of contextual, methodological, and geographic gaps. Contextually, the current CSA–income nexus studies in SSA skew toward rural small-scale farming contexts, with less focus on UA and peri-urban food systems. Again, many studies on CSA adoption tend to examine rural household surveys or general national datasets, widening the gap in understanding micro-level evidence from UA contexts, such as the ETH Municipality. Methodologically, existing studies rarely jointly model CSA adoption decisions with multiple economic (income and wealth) outcomes while accounting for selection bias in the UA context, particularly in the KZN Province, South Africa. Geographically, empirical evidence from urban farming systems in SSA, particularly South African metropolitan municipalities, remains sparse. Therefore, less is known concerning the association between CSA practices adoption and the income and wealth outcomes of SSUC farmers and their implications of food and nutrition security, particularly in South Africa’s rapidly urbanising and economically unequal society.

This study assesses the impact of CSA practices on SSUC farmers’ farm income in the ETH Municipality, KZN Province, with implications for food and nutrition security. This study adds to the existing body of knowledge on the linkages between CSA and income. Specifically, the study generates micro-level evidence on the nexus between CSA practices adoption and income/wealth outcomes among SSUC farmers in South Africa, with a focus on the ETH Municipality, and shows direct or indirect implications for food and nutrition security. Further, the study methodologically employs a conditional mixed-process framework to jointly model CSA practice adoption, farm income, and the wealth index, accounting for potential selection bias and endogeneity, which researchers have mainly overlooked in existing studies. Lastly, the study is specific to the ETH Municipality, a metropolitan and economically significant UA context that is largely under-researched. Therefore, the study context is intentional and provides insights and perspectives directly relevant to enhancing income, food, and nutrition security in urban areas of South Africa.

## 2 Conceptual framework

**Figure 1:**
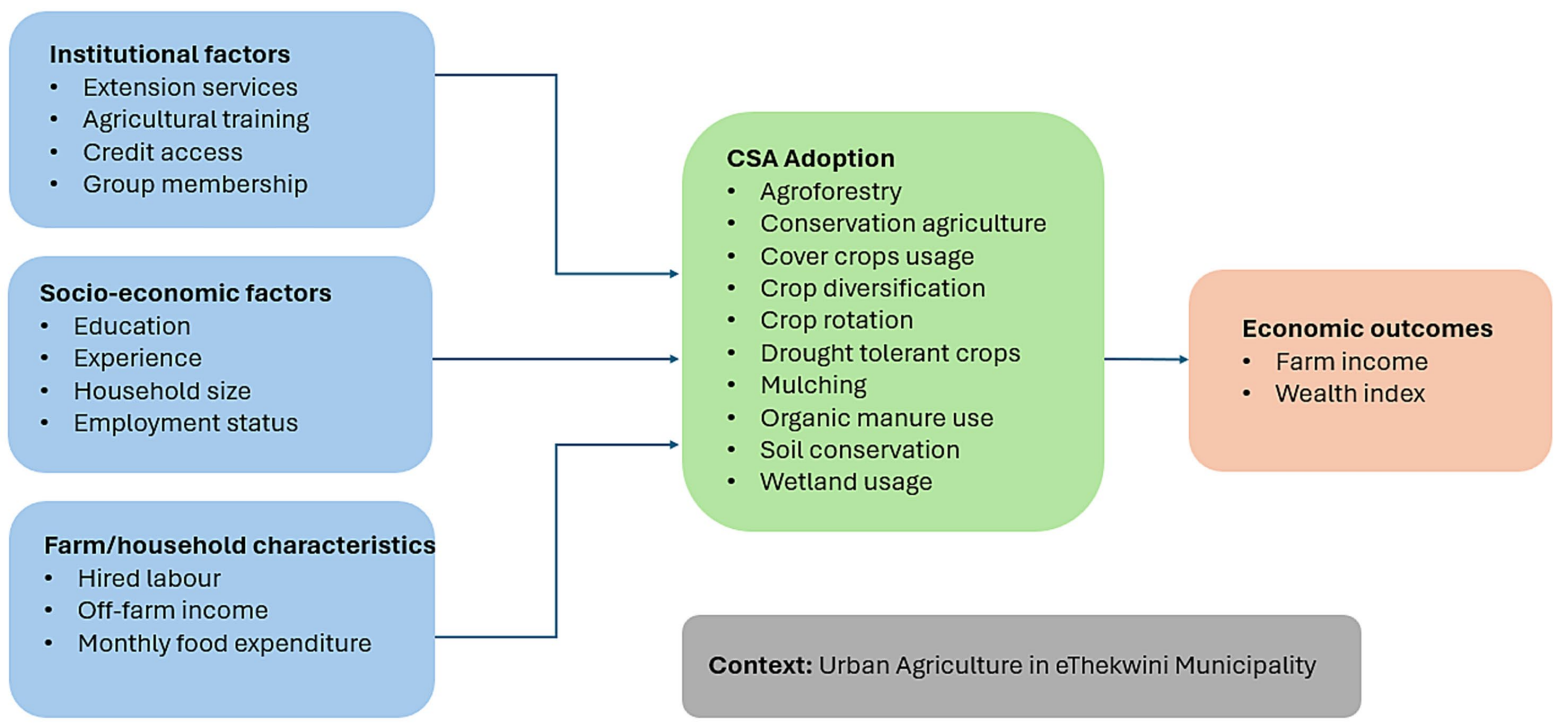
A conceptual framework of the association between climate-smart agriculture practices and small-scale urban farmers’ income security with implications for food and nutrition security. Source: Authors’ own conceptualisation based on literature, unpublished (2024).

This study’s conceptual framework depicts three aspects: (1) the socio-economic factors that influence CSA adoption and wealth (depicted by the wealth index), (2) the CSA practices adopted by SSUC farmers, and (3) the association with farm income and wealth with implications for food and nutrition security. In this paper, farm income is defined as the gross annual agricultural revenue from crop production by SSUC farmers, including the monetary value of crops sold and consumed over a 12-month reference period preceding the survey. Prevailing local market prices capture the full economic value of farm output. Therefore, farm income in this paper reflects gross farm revenue rather than net profit. The wealth index in this framework is asset-based (proxied by household and farm productive assets, including access to agricultural machinery, access to fertiliser inputs, capacity to purchase produce handling materials, use of agricultural production technologies, ownership and security of farm tools and equipment, and ownership of durable household assets) highlights socio-economic disparities that influence CSA practice adoption and income outcomes, which are essential to understanding the differential benefits between wealthier farmers and their counterparts (resource-poorer farmers). The influence of adopting a combination of CSA practices (A, CA, CD, CR, CC use, DT crops, M, OM use, W use and SC) is, therefore, expected to enhance SSUC farmers’ wealth and income security in the context of compounding factors that include human capital, socio-economic, and institutional factors (Figure 1). Figure 1 presents an original conceptual framework developed by the authors for this paper, guided by existing empirical and theoretical literature on CSA adoption and its associations with income and welfare outcomes. Socio-economic and institutional factors inform the decision to adopt a combination of CSA practices (37, 38). These explanatory factors determine the SSUC farmers’ ability to implement CSA practices effectively, thereby enhancing farm income, which is expected to directly or indirectly influence food and nutrition outcomes.

The study’s conceptual framework focuses on the central empirical relationship between CSA adoption (the primary explanatory variable) and farm income and the wealth index (the key outcome variables). In this framework, socio-economic characteristics (for example, education, household size, farming experience), institutional factors (such as access to credit, extension services, group membership), and farm-level characteristics condition CSA adoption among SSUC farmers. Simultaneously, these factors also shape farmers’ income and wealth outcomes with direct implications for food and nutrition security. The conceptual framework, therefore, shows CSA-income and wealth outcomes with food and nutrition implications, while recognising that socio-economic and institutional factors confound both adoption decisions and economic performance.

Central to the UA activities of SSUC farmers is the adoption of CSA practices to enhance farm income and wealth. Climate-smart agriculture is a globally recognised mechanism for adapting to and mitigating the effects of climate variability and change. The mitigation process reduces GHG emissions, whereas adaptation involves adjusting to actual or anticipated climate change impacts (39). Mitigation is universally applicable, unlike adaptation, which is location-specific (40). Adaptation is crucial in the UA sector, given its climate sensitivity (41). Therefore, adopting CSA is an essential adaptation strategy to improve the income, welfare, and livelihoods of UA communities in developing countries under climate change stress (42, 43). Climate-smart agriculture practices can help SSUC farmers increase productivity, thereby meeting growing food and nutritional demand and enhancing their income from surplus produce sales. This, in turn, leads to economic development and poverty reduction in urban settings (30, 34). Therefore, adopting CSA practices is expected to be positively associated with farm income and wealth, thereby contributing to the overall economic, food and nutrition well-being of SSUC farmers.

Understanding the influence of socio-economic and farm-level factors on the farmer’s income outcomes is crucial. Education is critical to adopting CSA practices and their impact on farm wealth and income. The rationale is that educated farmers are likely to comprehend and effectively implement CSA practices. Agricultural training, when relevant and high-quality, is expected to enhance SSUC farmers’ knowledge of CSA practices, thereby improving their income and wealth. Likewise, experienced farmers have a better understanding of CSA practices and are therefore more likely to see improvements in farm income from adopting them. Similarly, group membership facilitates knowledge sharing and collective action among SSUC farmers. Thus, membership in agricultural groups would be positively associated with farm income. Increased farm income and wealth are likely to directly or indirectly affirm the food and nutrition security of urban farmers.

Economic and labour factors are also crucial in implementing CSA practices to achieve financial benefits. Small-scale crop urban farmers with access to off-farm income could benefit from resources that are likely to intensify their adoption decisions regarding CSA practices, thereby increasing their productivity and ultimately positively impacting farm wealth and income. Large households contribute positively to farm wealth and income by providing labour or resources to adopt labour-intensive CSA practices. The effect of hired labour is inconclusive because, on the one hand, hired labour may mean appropriate and required skills are made available to implement the CSA practices optimally, yet on the other hand, the costs associated with hiring labour may outweigh the economic benefits of CSA practices adoption, thus negatively impacting the farm wealth and income of SSUC farmers. The employment status of an SSUC farmer contributes positively to farm income because employed farmers have access to improved resources, enabling them to invest in CSA practices, thereby enhancing farm productivity and income. Ultimately, the economic and labour dynamics associated with the adoption of CSA practices are expected to influence SSUC farmers’ food availability, stability, and nutritional outcomes.

Institutional factors, such as market arrangements, infrastructural endowments, extension services, and farm support policies, among others, affect farmers’ technology adoption (44). Access to critical resources, including credit and irrigation technology (infrastructure), is essential for improving farm income through the adoption of CSA practices. Access to credit alone may not be adequate to warrant income gains by SSUC farmers. However, tailored credit support mechanisms and adequate extension services are essential to successfully implementing CSA practices. Irrigation infrastructure provides SSUC farmers with reliable and sufficient water, likely to improve yields and directly increase farm income. Consequently, institutional factors are expected to shape farmers’ ability to improve food and nutritional security through consistent, nutrient-rich diversified food production arising from effective CSA adoption.

Climate-smart agriculture practices offer a pathway for enhancing farm income and wealth by improving resilience and productivity in the face of climate change-related stresses and challenges. Although the hypothesis is that adopting CSA practices would improve SSUC farmers’ income security and wealth, their association remains unclear. There is limited research on the association, linkages, or nexus between SSUC farmers’ adoption of CSA practices and income security, necessitating this study. However, the effective adoption of CSA practices and their association with farm income and wealth among SSUC farmers depend on various factors, including socio-economic, institutional and farm-level factors. Therefore, removing the challenges and constraints facing SSUC farmers would lead to the effective adoption of CSA and enhance income security, wealth, and food and nutrition outcomes.

While the current paper’s conceptual framework underscores that CSA is labour-intensive, it falls short in accounting for the opportunity cost of household labour. In urban settings, household members typically engage in off-farm employment or informal activities that are likely to yield higher returns than farm labour. Consequently, allocating more labour to CSA practices may increase farm income without necessarily increasing net welfare if it takes labour away from higher-paying off-farm activities. Therefore, researchers and policymakers should exercise caution in interpreting income gains associated with CSA adoption.

## 3 Materials and methods

### 3.1 Description and study area selection

**Figure 2:**
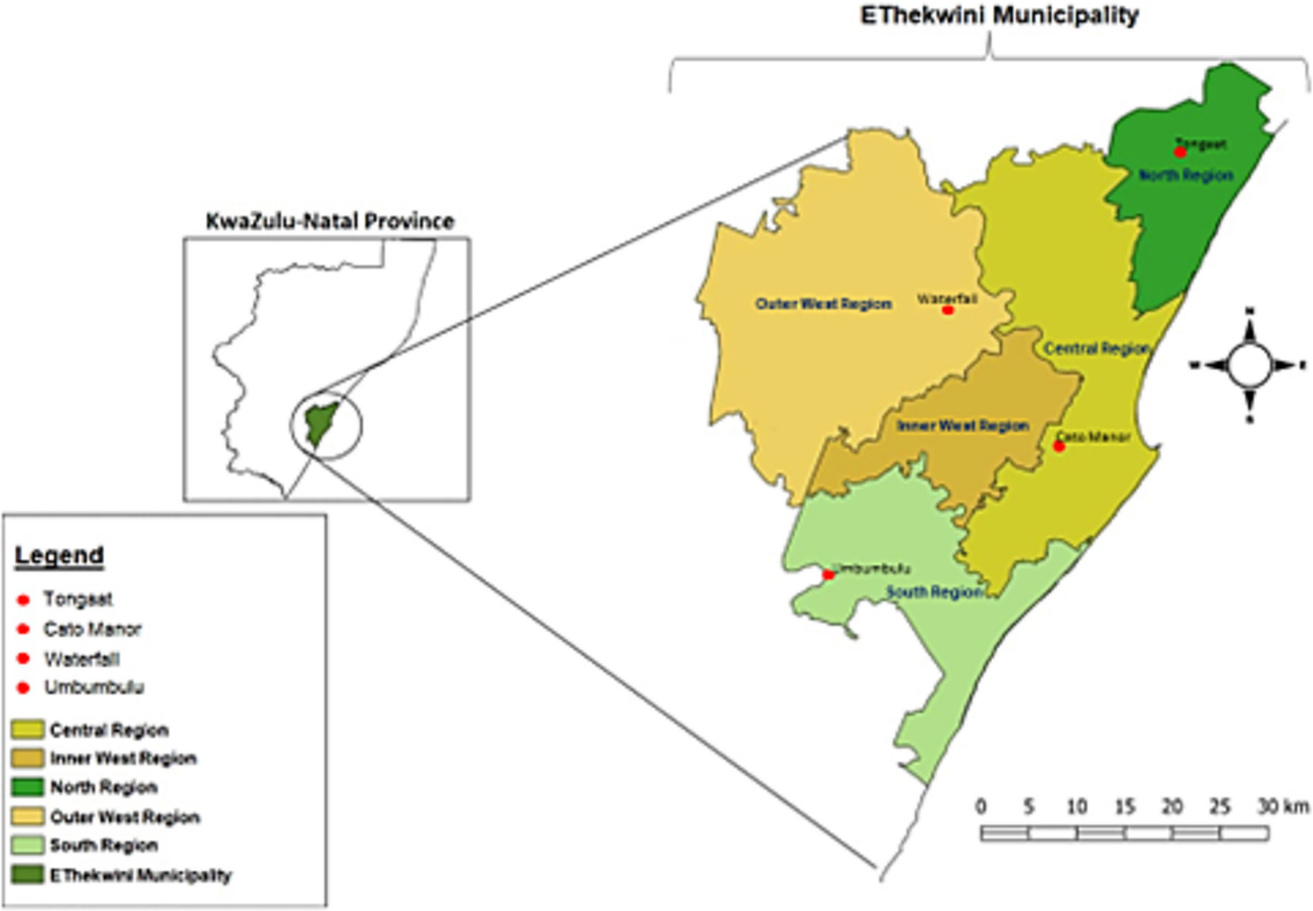
Map showing the study areas in eThekwini municipality, KwaZulu-Natal. Reproduced from Khumalo et al. (91), licensed under CC BY 4.0.

This researcher conducted the study in the ETH Municipality, KZN Province, South Africa, as depicted in Figure 2. The ETH Municipality is one of South Africa’s largest metropolitan areas after Johannesburg and Cape Town. The ETH Municipality’s geographic coordinates are 29.8120° S, 30.8039° E, spanning approximately 2,556 square kilometres.

The iLembe District borders the ETH Municipality to the north, the uGu District to the south, the uMgungundlovu District to the west, and the Indian Ocean to the east, giving it a strategic geographic position (45). Durban, the municipality’s primary urban hub, plays a pivotal role in the region. The province’s trademark agricultural commodity is sugarcane, with soybeans (7.9%) and maize (5.9%) being other major field crops (46). KwaZulu-Natal has the second-highest number of cattle in the country, accounting for 19% of the total (47). The province produces 28.3% of the nation’s milk, 11% of the country’s dairy producer-distributors and 15.2% of its milk processors live in this province (48). The selection of the ETH Municipality for this study was purposive, given its intensive agricultural activities and the significant concentration of SSUC farmers (49). The municipality’s coastal location exposes it to the impacts of climate change, such as rising temperatures and variable rainfall, providing an essential context for assessing the association between CSA practices aimed at boosting UA productivity, income, and resilience among urban farmers confronting climate and urbanisation pressures (50). The study adopted a multi-stage sampling method to identify distinct agroecological zones within the ETH Municipality, including Cato Manor Ward 29, Waterfall Ward 9, Tongaat Ward 62 and Umbumbulu Ward 109, each known for its UA capabilities. The region’s humid subtropical climate is favourable for various agricultural endeavours, characterised by hot summers, mild winters, average summer temperatures of 33 °C, winter temperatures of 16 °C, and an annual rainfall of 893 mm. Challenges such as reliance on rain-fed agriculture and ongoing land degradation threaten agricultural productivity, urban food security, and the livelihoods of urban farmers amidst increasing climate adversities (51). Furthermore, the growing population in the ETH Municipality underscores the importance of enhancing UA to support the expanding urban populace, highlighting the value and potential advantages of effective UA (52).

### 3.2 Research design

This study employed a quantitative cross-sectional design, enabling data collection at a specific point in time to assess the status of the phenomenon under investigation. This method is especially beneficial for studies aiming to minimise costs and maximise efficiency (53). Similar research by Daudu et al. (54), Jena et al. (55), and Fiawoo et al. (56) used a cross-sectional research design to examine the impact of CSA practices adoption on household farm income among small-scale farmers. A cross-sectional research design in the current study enabled a comprehensive analysis of the association between CSA practices adoption and farm income of SSUC farmers in the ETH Municipality. Employing a cross-sectional framework enabled a detailed examination of relevant variables, establishing a solid foundation for deriving informed conclusions about the association between adopting CSA practices and farm income and wealth in the UA setting (34).

### 3.3 Sample procedure and sample size determination

The study utilised a multi-stage sampling procedure, with purposive selection and snowball sampling applied at the final stage. Stage 1 involved the purposive selection of the study area, the KZN Province, and the ETH Municipality, due to the prevalence of UA and favourable climatic conditions conducive to UA. Subsequently, stage 2 of the multi-stage sampling involved selecting four specific wards within the municipality: Cato Manor Ward 29, Tongaat Ward 62, Umbumbulu Ward 109 and Waterfall Ward 9. The wards’ selection was justified by the significant UA activities in the wards and by their distinct climatic conditions that align with the study’s aim. Given the homogeneity of geographic and climatic conditions across these selected wards, the study hypothesised that these areas would not exhibit significant disparities in agricultural practices. The ETH Municipality does not have a complete sampling frame of SSUC farmers; therefore, stage 3 of the multi-stage sampling involved undertaking a multi-source mapping process. This stage involved engaging ward agricultural extension officers and community leaders (councillors) to identify eligible active SSUC farmers. Finally, stage 4 was the SSUC farmer selection. The study selected exclusively small-scale urban farmers actively engaged in crop farming within their wards. This process began with targeted identification of SSUC farmers in the area, followed by purposive and snowball (referral) sampling techniques, known for their efficiency and cost-effectiveness (57). The study applied snowball sampling as a final-stage augmentation only, using the initially identified SSUC farmers to refer additional farmers within the ward. To ensure adequate sample size representation, a Cochran’s formula was employed, as detailed in the following (Equation 1):

$$n_{\infty }=\frac{z^{2}p(1-p)}{e^{2}}$$ (1)

Where:

$n_{\infty }$ is the sample size.

$z^{2}$ is the standard error associated with the level of confidence.

*p* is the variability or standard deviation.

*e* is the ideal precision level (the margin of error).

*p* is the approximate proportion of the SSUC population.

Employing Cochran’s formula for a 95% confidence level and a 5% margin of error, a sample size of 384 SSUC farmers was determined (Equation 2):

$$n_{\infty }=\frac{{\left(1.96\right)}^{2}(0.5)(0.5)}{\left(0.05^{2}\right)}=384\;\text{farmers}$$ (2)

The study utilised multi-stage sampling, Cochran’s formula, and purposive sampling techniques to effectively navigate the complexities of the urban farming population, ensuring statistical accuracy and a comprehensive analysis. Typically, respondents in UA and CSA are small-scale farmers in SA who use family labour on plots smaller than 2 hectares for consumption and income generation. Per the sample size determination, the research initially targeted 384 SSUC farmers, aiming to select approximately 96 from each ward.

However, the data collection process exceeded initial expectations, yielding 412 respondents, distributed as follows: 101 from Cato Manor, Tongaat (110), Umbumbulu (98) and Waterfall (103). Given the Cochran’s sample size determination, the study initially targeted 384 SSUC farmers. During data collection, additional eligible farmers were recruited through snowball sampling, yielding a final sample of 412 respondents. The increase beyond the initial target reflects expanded participation rather than a response rate exceeding 100%, and all respondents met the predefined inclusion criteria. The snowballing technique facilitated the increased participation, extending beyond initial estimates. Ethically, it was essential to include all referred SSUC farmers to ensure inclusivity. Including additional respondents not only expanded the dataset but also improved the reliability of the results and provided more insights into the study’s aim. Therefore, the researcher deemed that oversampling would not negatively impact the research but instead enrich the understanding derived from the data.

Although the final sampling stage relied on purposive and snowball techniques, in the absence of a complete sampling frame for SSUC farmers in the ETH Municipality, the Cochran’s technique was employed as a benchmark to guide the minimum ideal sample size. The paper used Cochran’s formula not to claim population-level representativeness but to ensure sample size adequacy and statistical power rather than to imply probabilistic representativeness. This approach is consistent with applied empirical studies that combine non-probability sampling with formal sample-size benchmarks.

### 3.4 Data collection

This study employed a structured survey to gather primary data, explicitly designed to elucidate certain phenomena and test relational hypotheses within a well-defined framework (58). The survey comprised both closed- and open-ended questions targeting SSUC farmers in the ETH Municipality engaged in UA. The data collection method facilitated comprehensive descriptive and inferential analyses, which are critical for generating empirical evidence in support of the study’s objectives. To accommodate respondents’ language preferences, the researcher translated the questionnaire into isiZulu, a predominant language in the KZN Province, thereby enhancing clarity and comprehension.

A pilot study was conducted with 40 SSUC farmers from Tongaat, representing 10% of the target sample, to assess the questionnaire’s effectiveness (59). Although some researchers incorporate pilot data in the final analysis, this study excluded the pilot survey to minimise bias and uphold the integrity of the findings (60). Data collection took place between 29 May and 26 June 2023 and involved individuals engaged in UA at the household level. The researcher chose this period to collect data based on the previous season’s farming activities and metrics, ensuring uniformity and completeness across responses. The data encompassed socio-economic characteristics, including age, gender, household status, educational attainment, and economic activities, and provided insights into CSA practices and their association with income and wealth. Trained enumerators, proficient in ethical research practices and effective questioning techniques, administered the questionnaires to mitigate potential biases or misunderstandings (61). The researcher carefully timed data collection to avoid conflicts with local events, such as funerals, weddings, or social grant payout days, thereby ensuring maximum participation.

Necessary approvals were obtained, including ethics clearance from the University of KwaZulu-Natal (Protocol number: HSSREC/00005367/2023) and consent from the ETH Municipality’s Institute of Learning (MILE), before the commencement of the study. All respondents signed an informed consent form confirming their understanding of the research aims and procedures, thereby maintaining the study’s ethical standards.

### 3.5 Data management and analysis

The researcher meticulously organised and encoded data using Microsoft Excel 365 (Microsoft Corporation, Washington, United States). A comprehensive review ensured consistency, completeness, and the removal of any anomalies. After refining the dataset, it underwent analysis using Stata 18 (StataCorp, College Station, TX, United States). Multiple backups, including storage on a hard disk, an external hard drive, and cloud storage via OneDrive, with stringent security measures, safeguarded the data (62).

The study’s primary objective was to assess the association between CSA practices and farm income and wealth adopted by SSUC farmers in the ETH Municipality. Farmers employ a range of CSA practices and technologies to mitigate the impacts of climate change, with the adoption decisions interrelated with multiple factors (63). The conditional mixed process (CMP) model provides a comprehensive analytical framework suitable for investigating complex relationships among multiple interdependent variables. The CMP enables the simultaneous modelling of multiple equations that may be interdependent, reflecting the reality that decisions (such as the adoption of various CSA practices) are often not made in isolation but are made jointly with other decisions and factors (64). This interdependence is crucial in accurately reflecting the decision-making process in agricultural settings, where considerations account for multiple practices rather than operating independently. Various hypothesised explanatory factors, informed by the literature, served as predictor variables.

Endogeneity in agricultural technology adoption studies arises from unobserved heterogeneity, simultaneity bias, and measurement errors, which can lead to biased estimates if not properly addressed (65). Various methods have tried to mitigate this issue, including the instrumental variable (IV) approach, which utilises external instruments such as distance to extension services; however, finding valid instruments remains challenging (66). The control function approach corrects for omitted variable bias by incorporating residuals from an auxiliary regression into the main model (67). Simultaneous equation models (SEM) and CMP estimation jointly model interdependent decisions while accounting for correlated error terms, making CMP particularly useful for analysing CSA adoption (68). Although some studies utilise panel data techniques to control for unobserved heterogeneity, these methods are unsuitable for this study, given its cross-sectional design (69). Given these factors, the CMP model effectively captures the interdependence among CSA adoption decisions, addresses endogeneity concerns, and enhances the reliability of estimates of the relationships between CSA adoption and farm income.

#### 3.5.1 Descriptive statistics

The study employs descriptive analysis to examine the demographic and socio-economic profiles of SSUC farmers and the CSA practices implemented in the ETH Municipality. This descriptive analysis includes frequency distributions (presented as percentages) and measures of central tendency, including means, ranges, and standard deviations. The study findings are displayed in graphs and tables to provide clear visual and quantitative summaries. Asante et al. (63) and Tanti et al. (70) have demonstrated the value of descriptive statistical methods in assessing the association between adopting CSA practices and farm income. In the current study, descriptive analysis highlights prevailing trends and lays the groundwork for more detailed inferential analysis, ensuring a comprehensive understanding of the dynamics at play.

#### 3.5.2 Wealth index construction

The study computed a household wealth index using principal component analysis (PCA) based on asset ownership and housing characteristics. The wealth index is a composite of household and farm productive assets, including access to agricultural machinery, access to fertiliser inputs, capacity to purchase produce-handling materials, use of agricultural production technologies, ownership and security of farm tools and equipment, and ownership of durable household assets. The asset variables were binary and were standardised before principal component analysis to ensure comparability. A high Kaiser–Meyer–Olkin (KMO) (0.905) confirmed the suitability of the PCA statistic and a significant Bartlett’s test of sphericity (*p* < 0.001). Following the Kaiser criterion, the first principal component (eigenvalue > 1), which explained 54.4% of the total variance, was retained as the wealth index. The resulting component scores were subsequently normalised to have a mean of zero and a standard deviation of one for ease of interpretation in the CMP model. A higher index value indicated greater household wealth. The results were robust to alternative scaling approaches, including a simple additive index. Appendix 1 details the component loadings and summary statistics.

#### 3.5.3 Conditional mixed process model

A CMP model was used to assess the association between CSA practices and SSUC farmers’ farm income and wealth in the ETH Municipality. Given the potential for endogeneity in adopting CSA practices, a robust methodological approach is necessary to ensure the validity of the estimated effects. The primary focus of this analysis was to estimate the relationship between CSA practice adoption and farm income, with the individual SSUC farmer as the unit of analysis. The following structural equation specifies the relationship (Equation 3):

$$y_{i}=\alpha_{0}+\alpha_{1}\mathrm{CS}A_{i}+X\prime_{i}\alpha_{2}+\delta_{w(i)}+\varepsilon_{i}\;$$ (3)

Where:

-
$y_{i}$ represents the farm income or wealth index of farmer i .
-
$\mathrm{CS}A_{i}\;$ is a binary variable representing the adoption of CSA practices by an SSUC farmer i (1 if adopted, 0 otherwise).
-
$X_{i}\;$ is a vector of control variables encompassing household and farm-level characteristics, hypothesised to influence income.
-
$\delta_{w(i)}\;$ captures unobserved heterogeneity specific to each location, assumed to be uncorrelated with $X_{i}$ .
-
$\varepsilon_{i}$ is the idiosyncratic error term, assumed to follow an independent and identically distributed normal distribution with mean zero and unit variance.

While CSA adoption varies by intensity and diversity, the paper used a binary indicator (CSA adopter versus non-adopter) as the primary variable to capture the income and welfare outcomes beyond a minimum adoption threshold. Here, farmers below the mean CSI threshold of 6 were classified as low-level adopters (non-adopters) while those above the mean were classified as adopters. This specification aligns with the CMP framework for modelling joint decisions to adopt CSA practices and their effects on economic outcomes, while addressing selection bias. To account for variation in intensity and diversity, a composite score index (CSI) of CSA practices adopted by SSUC farmers was computed using PCA for robustness checks. Therefore, this approach ensured the internal validity of the CSA binary measure.

A critical methodological concern in this study was the potential endogeneity of adopting CSA practices. Endogeneity may arise from several sources, including omitted variables, reverse causality, or measurement errors. Failure to account for this endogeneity could lead to biased and inconsistent parameter estimates, particularly in assessing the true impact of CSA practices adoption on income. In this study, to address the problem of endogeneity, CSA practices adoption was modelled as an endogenous variable using the following latent variable framework in Equation 4:

$$\mathrm{CS}A_{i}^{*}=\beta_{0}+Z\prime_{i}\gamma +X\prime_{i}\beta_{2}+\delta_{w(i)}+v_{i}$$ (4)

Where:

-
$\mathrm{CS}A_{i}^{*}$ is the latent propensity for an SSUC farmer 𝑖 to adopt CSA practices.
-
$Z_{i}$ is a vector of instrumental variables influencing the adoption of CSA practices, but it is not directly related to farm income or wealth index.
-
$X_{i}$ includes observable household and farm characteristics such as farm size, access to irrigation infrastructure, farmer education and other factors.
-
$\delta_{w(i)}$ is the unobserved heterogeneity specific to each location that affects the adoption of CSA practices, but it is assumed to be uncorrelated with control variables hypothesised to influence farm income.
-
$v_{i}$ represents the error term.

The study employed an instruments-and-identification strategy, $Z_{i}$ , developed from information from the Department of Agriculture and farmer-cross-referenced sources, namely: distance to the nearest agricultural extension support office (kilometres), exposure to a CSA demonstration or training within the previous 12 months, and membership in a CSA-producer group. The first-stage regression confirms the relevance of the instruments, as distance to extension services, exposure to a CSA demonstration or training, and membership in a CSA-producer group significantly influence CSA adoption by collectively enhancing CSA technical information, lowering the transaction costs of adoption, and thus promoting wider CSA uptake. However, when included in the income/wealth equation, they are not statistically significant, suggesting they do not directly affect household income security. These instruments satisfy only the CSA adoption equation and were deliberately excluded from the outcome equation to satisfy the required exclusion restrictions. Conceptually, they provide information, but no material income benefits, thereby meeting theoretical exclusion criteria. The researchers assessed instrument validity by reporting the joint significance of the instruments in the CSA adoption equation (*χ*^2^ test) (Appendix 2), confirming strong instrument relevance and the estimated statistically significant *ρ*, and a likelihood-ratio test of ρ = 0 indicating endogeneity in CSA adoption. Based on the likelihood-ratio test of ρ = 0, the paper rejects the null hypothesis, justifying the use of the CMP framework. The researchers also conducted sensitivity checks by replacing or omitting instruments and by re-estimating the models using alternative CSA adoption measures (binary threshold-based adoption as the main specification, and count- and index-based measures as robustness checks). The diagnostic tests are reported in Appendix 2. Falsification and sensitivity analyses confirm instrument relevance and exogeneity, while additional robustness checks using alternative CSA adoption measures yield qualitatively consistent results.

Given the endogeneity of CSA practices adoption, traditional estimation techniques such as Ordinary Least Squares (OLS) may yield biased results. The CMP model mitigates endogeneity (71). This model enables the joint estimation of the income and CSA adoption equations, thereby accommodating potential correlation between their error terms. The CMP model is well-suited for this study, as it enables the estimation of recursive models with mixed processes. By allowing for cross-equation correlations in the error terms, the CMP model corrects for selection bias induced by unobserved factors that may simultaneously influence both CSA practices adoption and farm income. This method has been validated in similar studies (72, 73), providing support for its application in this study.

#### 3.5.4 Description of the explanatory variables used in the conditional mixed processes model

Table 1 summarises the explanatory variables included in the CMP model to assess the association between CSA practices adoption and farm income and wealth among SSUC farmers in the ETH Municipality. The study hypothesises that adopting CSA practices will significantly increase the farm wealth index and income. Explanatory factors expected to influence farm wealth and income positively include principal economic activity (farming), adequate agricultural training, agricultural group membership, access to irrigation infrastructure, large household size, educated SSUC farmers, household income, farming experience and proximity to the farming site (63, 74, 75). These factors directly correlate with access to resources, knowledge, and opportunities necessary to improve farm productivity and income. For instance, farmers who primarily engage in farming as their main economic activity, have access to irrigation infrastructure, or have received adequate agricultural training are better positioned to adopt CSA practices, leading to improved crop yields and higher incomes. Similarly, higher education levels, larger household sizes (providing labour), higher household income, membership to agricultural groups, and farming experience contribute positively to adopting CSA practices by providing the skills, financial stability, and workforce needed to manage farming practices effectively (76–78). Additionally, shorter distances between the home and the farm site enable farmers to manage their operations more efficiently, encouraging the adoption of CSA practices and increasing farm income.

**Table 1:** Explanatory variables to assess the association between the adoption of climate-smart agriculture practices and farm income.

Variable	Description and measurement (type)	Expected outcome (+/−)	Supporting literature
Adoption of CSA practices	Whether the SSUC farm adopts CSA (non-adoption of CSA = 0; adopts CSA = 1) (dummy)	+	(115, 116)
Gender	SSUC farmer’s gender (female = 0; male = 1) (dummy)	−	(99, 117)
Marital status	Marital status of the SSUC farmer (single = 0; wedded (married, divorced, widowed) = 1) (dummy)	+/−	(118, 119)
Principal economic activity	SSUC farmer main economic activity (formal employment = 0; farming = 1) (dummy)	+	(92, 120)
Agricultural training received	Whether the SSUC farm has received agricultural training (no = 0; yes = 1) (dummy)	+	(121, 122)
Access to irrigation	Whether the SSUC farmer had ready access to irrigation technology (no = 0; yes = 1) (dummy)	+	(123, 124)
Age	Age of the SSUC farmer in years (continuous)	+/−	(125, 126)
Education (schooling years)	Number of years of formal schooling by the SSUC farmer (continuous)	+	(92, 121)
Household size	Number of members in the SSUC farmer’s household (continuous)	+	(124, 127)
Hired labour	Whether the SSUC farmer hired labour (no = 0; yes = 1) (dummy)	−	(128, 129)
Part-time labourers from the household	Number of part-time labourers from household (continuous)	−	(130, 131)
Household income	Total yearly household income (continuous)	+	(132, 133)
Off-farm income	Total yearly off-income (continuous)	+/−	(134, 135)
Monthly expenditure on food items (ZAR)	Monthly expenditure on food items (continuous)	−	(136, 137)
Farming experience	Number of years of farming experience by the SSUC farmer (continuous)	+	(138, 139)
Average distance to the farming site/ farm	The distance from home to the farm site in kilometres (continuous)	+	(134, 140)

± Denotes the direction of influence.

On the other hand, explanatory factors expected to influence farm wealth and income negatively include hired labour, part-time household labourers, and monthly food expenditures. The predicted outcome suggests that the costs associated with hired and part-time household labour may reduce farm efficiency, limiting the adoption of CSA practices and thus lowering farm income. Similarly, higher monthly food expenditures may divert financial resources from farm investment, reducing the likelihood of adopting CSA practices and thereby negatively affecting farm income.

Variables expected to have mixed influences on farm wealth and income include marital status, age, and off-farm income. These variables reflect the complex socio-economic factors that influence a farmer’s decision-making process regarding the adoption of CSA practices. For example, married farmers may benefit from spousal support, which could increase their ability to adopt CSA practices; however, they may also face additional financial responsibilities that limit their investment in farming. Similarly, younger farmers may be more open to adopting new farming technologies. In contrast, older, more experienced farmers possess adequate expertise and resources that may aid in the effective adoption and implementation of CSA practices. At the same time, older farmers may prefer traditional farming methods, which could hinder the effective adoption and implementation of CSA. The impact of off-farm income is also mixed: it can provide additional financial resources for CSA investment or divert attention from farming activities, depending on the farmer’s level of commitment to agriculture.

Some explanatory variables, specifically household food expenditure and hired labour, could be jointly estimated with farm income. However, in this paper, household food expenditure and hired labour were treated as conditional exogenous controls, that is, as household and production characteristics rather than CSA adoption outcomes. Therefore, in this paper, the researchers interpreted these variables as proxies for baseline consumption needs and production or labour-management choices, rather than as causal effects, to reduce omitted-variable bias.

## 4 Results and discussion

**Figure 3:**
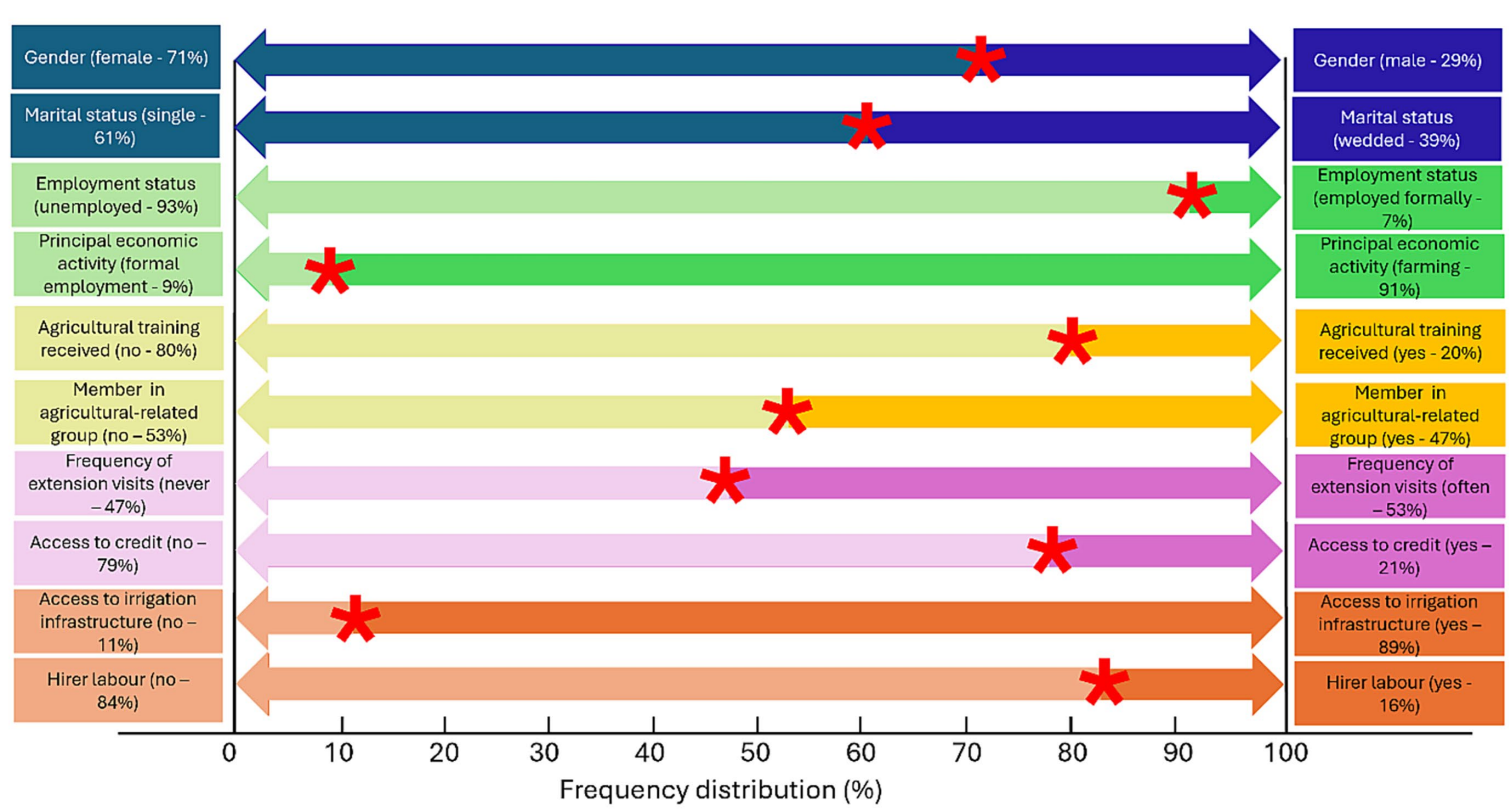
Categorical distribution of socio-economic and farm characteristics of the sampled small-scale urban crop farmers in eThekwini municipality. Source: Survey data (2023).

Figure 3 shows the frequency distribution of the categorical socio-economic and farm characteristics of the sampled SSUC farmers in the ETH Municipality. The results indicate a higher prevalence of female SSUC farmers (71%) than males, suggesting a female-dominated small-scale agriculture in the ETH Municipality. Female farmers, especially in small-scale contexts, often face unique challenges, such as limited access to land, credit, and agricultural inputs, compared to their male counterparts, which influences their overall productivity and farm income. Duvivier et al. (79) observed that farm income for male-headed farms was almost 2.5 times greater than for female-headed farms.

### 4.1 Demographic characteristics of the sampled small-scale urban crop farmers in eThekwini municipality

Marital status reveals that most (61%) SSUC farmers were single. Single farmers have complete autonomy in decision-making, which may enable them to quickly adopt CSA practices without consulting a spouse or other household members (80). This autonomy could positively impact farm income if CSA practices improve farm output. A significant proportion (93%) of SSUC farmers reported being unemployed, indicating a high unemployment rate among the sampled SSUC farmers. The results suggest that unemployed farmers may have the time to invest in labour-intensive CSA practices, which could improve farm productivity and increase farm income (35).

Most (91%) SSUC farmers primarily engaged in UA farming as their principal economic activity, while the remainder relied on formal employment. The findings indicate a high reliance on UA as a livelihood strategy. Farmers who rely solely on UA for their livelihood may face greater economic pressure to increase productivity and farm income from their UA activities. Poudel et al. (81) emphasise that adopting CSA practices is crucial in improving farm resilience, increasing yields, and ensuring sustainable farm income. Most (80%) SSUC farmers in the ETH Municipality had not received specialised agricultural training. The remaining few (20%) had attained some form of agricultural training, ranging from certificates/ degrees to unaccredited short courses. The findings suggest that SSUC farmers may be less knowledgeable about advanced or modern farming techniques, including CSA practices, and are likely to continue using traditional, less efficient farming methods, which may result in lower productivity and farm income. Mpala and Simatele (82) emphasised that training is pivotal in effectively implementing CSA programmes for small-scale farmers.

Nearly half (47%) of the sampled SSUC farmers belonged to a farming-related group, with the remainder slightly above half (53%) not associated with any agricultural-related group or association. Farmers’ organisations provide a platform for farmers to exchange knowledge, skills, production experiences, and social capital, thereby supporting the adoption of CSA practices (76). The findings suggest that SSUC farmers who were members of farming groups were more likely to be exposed to CSA practices and other resource-saving methods, potentially leading to higher adoption rates that could positively impact their farm income through improving crop yields, reducing input costs, and enhancing resilience to climate variability.

Access to extension services remains challenging, as a substantial proportion (47%) of SSUC farmers have never received visits from extension agents or workers. However, slightly above half (53%) of SSUC farmers reported that extension workers visited them frequently and seasonally. Anang and Apedo (83) reveal a positive relationship between extension services and farm income among small-scale farmers. The findings suggest SSUC farmers who regularly receive extension service visits are likely to have better access to knowledge, adopt a combination of CSA practices more quickly, and improve their farm income. Most (79%) of the sampled SSUC farmers lacked access to credit. The results suggest that SSUC farmers in ETH Municipality may struggle to adopt CSA practices, even if they are aware of their benefits, due to the initial upfront costs. Without credit, farmers may be unable to invest in CSA practices, reducing their capacity to increase yields or diversify crops and limiting their ability to boost farm income (84).

A higher proportion (89%) of SSUC farmers in the ETH Municipality had access to irrigation infrastructure (they irrigate crops using municipal water, though it is illegal). While SSUC farmers in ETH had high access to irrigation infrastructure, using municipal water without formal authorisation introduces a critical institutional risk, exposing them to service disruptions and potential future restrictions, likely to undermine the sustainability of observed production and income gains from CSA practices investment. The findings suggest that SSUC farmers reduce their reliance on rain-fed agriculture, making it easier for farmers to adopt techniques that require consistent water availability. Irrigation infrastructure enables farmers to grow crops more consistently and reliably, including during dry/ off seasons or under drought conditions, leading to more stable production and higher yields (85). A higher (84%) proportion of SSUC farmers did not hire labour. This finding proves that most SSUC farmers rely on their own or family labour for their UA activities. This situation may reflect financial constraints that limit scaling up farming operations or a preference for self-management among SSUC farmers. However, reliance on personal or family labour could imply limited skills to increase productivity and UA scalability, thereby affecting their farm income.

Table 2 presents the continuous distribution of socio-economic and farm characteristics among SSUC farmers in the ETH Municipality. The age of SSUC farmers in the ETH Municipality spanned from 28 to 80 years, with an average of 55 years. Ninsheka et al. (86) noted that small-scale urban farmers are generally older individuals. Older farmers may be more risk-averse and less likely to adopt new technologies or practices due to familiarity with traditional methods or limited access to new information (87). Ndung’u et al. (88) observed that older farmers may be less inclined to invest in long-term improvements, such as CSA practices, due to shorter planning horizons, which could limit income growth over time. On the other hand, older farmers may possess more experience and social capital, which could positively influence decision-making and access to resources that stabilise or increase income (89). Table 2 indicates that SSUC farmers in ETH Municipality have an average of 8 years of schooling, ranging from no formal education to 18 years. The findings suggest that SSUC farmers possess basic education that can enable them to understand the.

**Table 2:** Continuous distribution of socio-economic and farm characteristics of the sampled small-scale urban crop farmers in eThekwini municipality.

Variable	Mean	Min	Max	SD
Age	54.607	28	80	11.898
Education (years)	8.228	0	18	4.230
Household size	8.289	1	19	3.450
Farm income (annual) (ZAR)	155563.80	12,600	361,800	60903.44
Off-farm income (yearly) (ZAR)	17132.87	8,000	117,500	10650.80
Monthly expenditure on food items (ZAR)	3624.77	650	7,000	1494.77
Number of part-time labourers from the household	1.803	0	7	1.270
Average distance to farming site/ farm (km)	2.88	0.1	12	2.29
Farming experience	19.388	5	52	10.153

ZAR, Max, Min and SD stand for South African Rands, Maximum, Minimum and Standard deviation, respectively. Source: Survey data (2023).

benefits of CSA, access to relevant training, and utilise modern technologies. Farmers with some education would likely have better access to information, extension services, and financial opportunities, which can improve farm productivity and income (90).

The results show that SSUC farmers in ETH Municipality have relatively large household sizes, with an average of 8 members and a range from 1 to 19. A large household size is advantageous to farmers, as it can provide additional labour, which is often crucial for implementing labour-intensive CSA practices, such as M, CR, or A. Therefore, the availability of family labour may allow farmers to adopt labour-intensive CSA practices without incurring extra costs for hired labour. While more household members may contribute to farm labour, they could also increase household consumption needs, reducing the surplus produce available for sale and limiting the ability to save or invest in farm improvements to derive economic benefits.

Farm income is crucial for reinvesting in UA activities. SSUC farmers in the ETH Municipality had annual farm incomes ranging from ZAR 12600 to ZAR 361800, averaging ZAR 155563.80. This income level, though lower compared to the South African national average wage of ZAR 26,032 per month suggests a relatively stable financial status that could support initial investments in full-scale CSA practices. However, farmers near the lower end of the spectrum may face financial constraints, limiting their ability to adopt CSA practices without external support or credit. Farmers with higher incomes may benefit from improved access to markets, technologies, or farm management practices, enabling further investments in CSA practices to yield greater economic gains (91).

The yearly off-farm income results ranged from ZAR 8000 to ZAR 117500, averaging ZAR 17132.87. The findings suggest that SSUC farmers with additional sources of revenue outside farming are generally more financially capable and may be more likely to adopt CSA practices (92). Off-farm income provides an additional source of revenue that can supplement farm income. Off-farm income diversification helps mitigate the risks associated with agriculture, particularly during crop failures or climate-related shocks (93). For farmers earning higher off-farm incomes, the additional funds could help to stabilise household finances and reinvest in their farming operations, potentially leading to higher farm income by adopting more efficient and productive CSA practices (94).

The findings show that SSUC farmers’ monthly food expenditures in the ETH Municipality ranged from ZAR 650 to ZAR 7000, with an average of ZAR 3624.77. The results reveal a relatively high average monthly food expenditure, indicating that SSUC farmers allocate a significant portion of their household income to food. This situation could limit the financial flexibility for investment in CSA practices. Belay et al. (34) observed that farmers with higher food expenditures may prioritise meeting short-term to immediate household food needs over long-term investments in CSA practices that could enhance their income security.

The sampled SSUC farmers in ETH Municipality had part-time labourers from households ranging from none (0) to a maximum of 7 members, with an average of approximately 2 members available for part-time farm work. The findings suggest a low labour force available from the household to support their farming operations. The dependency on limited household labour can limit the scalability and modernisation of labour-intensive CSA farming operations in UA.

The SSUC farmers travelled an average distance of 3 km to farming sites (open or vacant spaces) to practice their UA activities from their residences within the specific wards in the ETH Municipality. The average distance of 3 km to farming sites can be challenging, given the elderly population of SSUC farmers in the ETH Municipality. A distant farming site increases transportation costs and time delays, making it harder to implement CSA practices effectively. Debela et al. (95) observed a negative relationship between farm distance and the adoption of CSA practices, such as water harvesting and intercropping, with these practices being less frequent at greater distances. Long distances make it difficult to maintain CSA practices, reducing their effectiveness and potential income benefits (96).

The SSUC farmers in ETH Municipality have considerable farming experience, ranging from 5 to 52 years, with an average of 19 years. Experienced farmers will likely have a deep understanding of local agricultural conditions, climate patterns, and traditional farming techniques. This extensive farming experience could facilitate the adoption of CSA practices, as experienced farmers would likely possess the knowledge and skills to implement them, thereby contributing to stable or higher farm income (97).

### 4.2 Climate-smart agriculture practices implemented by small-scale urban crop farmers in the eThekwini municipality

**Figure 4:**
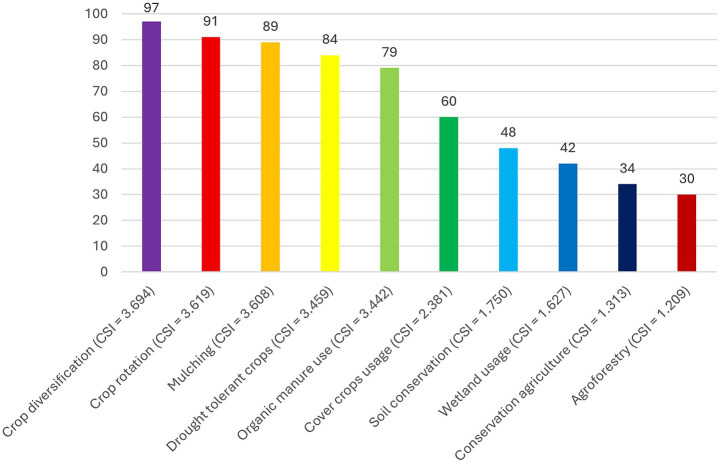
Adoption of climate-smart agriculture practices in eThekwini municipality.

The study profiled the specific CSA practices adopted by SSUC farmers in the ETH Municipality. The SSUC farmers adopted CSA practices that include A, CA, CD, CR, CC use, DT crop varieties, M, OM use, W use, and SC. As already explained in section 3.5, the CSA practices were measured using a binary indicator (adopters and non-adopters), variation in adoption intensity was accounted for through a CSI (ranging from 0 to 10), and a normalised composite index was generated through PCA. Descriptive statistics present the CSA adoption rate of SSUC farmers in the ETH Municipality. Figure 4 summarises the level of use of CSA practices among SSUC farmers in the ETH Municipality. The CSI reflects the relative adoption and effectiveness of the different CSA practices used by these farmers. Crop diversification ranked highest among CSA practices, with a CSI of 3.694, followed by CR (3.619), M (3.608), DT crops (3.459), and OM application (3.442). The farmers in the study area widely adopt CSA practices that offer immediate benefits, low complexity, and low cost, reflecting the resource constraints faced by small-scale farmers (98). Other CSA practices adopted by farmers include cover cropping (2.381), soil conservation (1.750), wetland usage (1.627), conservation agriculture (1.313), and agroforestry (1.209), which were ranked lowest in that order. The lower preference for these practices may be due to implementation-related challenges, such as greater complexity, higher initial investment costs, and longer time required to realise benefits (99). Consequently, addressing these barriers is essential to promote the wider adoption and scaling up of CSA practices among small-scale farmers.

### 4.3 The association between climate-smart agriculture practices adoption and farm income and wealth of small-scale urban crop farmers in eThekwini municipality, with implications for nutrition

Table 3 presents the combined analysis of the relationship between adopting CSA practices and farm income, as well as the latent propensity to adopt CSA. In addition to the estimated association between income and welfare, the CMP framework jointly models the determinants of CSA adoption. The first-stage adoption equation shows that farming experience, education level, household size, and access to institutional support significantly increase the likelihood of adopting CSA practices. On the other hand, labour constraints and participation in non-farm activities reduce the propensity to adopt CSA. The results underscore that human capital and farm-specific experience are critical determinants of CSA adoption by SSUC farmers. The joint estimation allows for the consideration of endogeneity biases arising from selection biases in CSA practice adoption and from the relationship between wealth index and farm income. The transformation of the correlation coefficient (/atanhrho_12) indicates the transformed correlation between the errors of the two dependent variables (farm wealth and income). The estimated correlation coefficient (𝜌12 ≈ −0.24) is statistically.

**Table 3:** Determinants of climate-smart practices adoption on farm wealth and income using the conditional mixed process model.

Variable	Wealth index			Farm income		
	Coef.	Std. err	*p*-value	Coef	Std. err	*p*-value
CSA adoption (yes/no)	0.386	0.099	0.000***	139.711	54.23	0.011***
Age (years)	−0.003	0.004	0.431	−64.415	63.437	0.310
Gender (male/ female)	−0.114	0.091	0.210	2028.888	1323.417	0.125
Marital status (wedded/ single)	−0.185	0.043	0.000***	−206.175	633.953	0.745
Education (schooling years)	0.138	0.016	0.000***	869.164	226.218	0.000***
Agricultural training received (yes/ no)	−0.458	0.060	0.000***	−1813.384	874.340	0.038**
Principal economic activity (formal employment/ farming)	−0.432	0.318	0.174	−10,400	4636.729	0.025**
Hired labour (yes/ no)	−0.827	0.090	0.000***	−21,000	1319.389	0.000***
Number of part-time labourers from the household	−0.619	0.084	0.000***	−21,200	1229.214	0.000***
Household size	0.608	0.085	0.000***	21824.010	1236.017	0.000***
Farming experience	0.020	0.005	0.000***	286.260	75.137	0.000***
Distance to farming site (kilometres)	0.092	0.019	0.000***	248.981	270.606	0.358
Access to irrigation infrastructure (yes/ no)	−0.404	0.132	0.002***	−2659.478	1929.260	0.168
Monthly expenditure on food items (ZAR)	0.000	0.000	0.000***	0.753	0.047	0.000***
Off-farm income (yearly) (ZAR)	−0.563	0.100	0.000***	−10,200	1461.587	0.000***
Employment status (employed/ unemployed)	1.914	0.493	0.000***	46,500	7191.853	0.000***
Access to credit (yes/ no)	0.045	0.101	0.654	2282.107	1467.165	0.120
Membership in an agricultural-related group (yes/ no)	−0.142	0.093	0.127	2392.026	1357.348	0.078*
-const	7.257	1.546	0.000	146,000	22561.440	0.000
/atanhrho_12	−0.247	0.035	0.000			
/lnsig_1	9.342	0.035	0.000			
/lnsig_2	0.332	0.049	0.000			
rho_12	0.320	0.044	0.000			

The asterisks *, **, and *** show significance at 10, 5 and 1% levels, respectively, with econometric data modelling generated through stata version 18.

significant, validating endogenous selection in CSA adoption decisions and the CMP estimation strategy, suggesting that unobserved factors jointly influence CSA adoption, farm income, and wealth. The significance of the correlation parameter, the rejection of the likelihood-ratio test of independence, and the strong joint relevance of the instruments confirm that the instruments are suitable and that endogeneity is addressed meaningfully within the CMP framework.

Therefore, this analysis shows that the transformation of the correlation coefficient indicates that the CMP model accurately captures the interdependence between wealth and income, making joint estimation more suitable. The CMP model results in Table 3 show that CSA practice adoption, education, household size, farming experience, and employment status were significant and positively associated with farm wealth and income, while monthly food expenditure and membership in an agricultural-related group were marginally significant and positively associated with farm income. In contrast, agricultural training received, hired labour, the number of part-time labourers, and off-farm income all negatively affected farm wealth and income. Additionally, access to irrigation infrastructure and the principal economic activity (formal employment) negatively affected farm wealth and income, respectively. Marital status, membership in agricultural groups, and principal economic activity had a significant negative impact on farm wealth.

Table 3 shows that CSA practices adoption has a positive and statistically significant influence on the wealth index [coefficient (*β*) = 0.386, *p*-value = 0.000] and farm income (*β* = 139.711, *p*-value = 0.011) at a 1% level. The results suggest that adopting CSA practices can improve SSUC farmers’ wealth index and farm income in the ETH Municipality. The CSA adoption coefficient of 139.711 suggests that CSA practice adopters earn, on average, approximately ZAR 139 (about 1%) more in annual gross farm income than non-adopters. While this variable is statistically significant, the coefficient relative to the sample mean income of ZAR 155563.80 is minor, and implies a modest income association rather than a transformative income gain. However, for lower-income or resource-constrained SSUC farmers, an increase of approximately ZAR 140 could represent a meaningful marginal gain, alongside non-monetary gains such as improved yields and reduced climate risk. Urban farmers who adopt CSA practices are likely to increase their resilience and enhance productivity through sustainable agriculture, which contributes to long-term asset accumulation, including increased farm profitability and higher farm income. From a nutrition perspective, even modest income gains can enhance food access and dietary diversity by expanding access to a range of nutritious foods through the adoption of CSA practices.

Education empowers farmers with better decision-making skills, more efficient farming practices, or greater access to markets and financial resources. Education had a statistically significant positive influence on the wealth index (*β* = 0.138, *p*-value = 0.000) and farm income (*β* = 869.164, *p*-value = 0.000) at a 1% level of significance. The findings suggest that education significantly enhances the farm wealth and income of SSUC farmers in the ETH Municipality. Mujeyi et al. (100) found a positive association between education and agricultural income among small-scale farmers in Zimbabwe. Higher levels of education can have positive nutrition implications, stemming from better decision-making by high-income households to invest in diversified, higher-quality diets and improved food security.

Surprisingly, agricultural training had a statistically significant negative influence on the wealth index (*β* = −0.458, *p*-value = 0.000) and farm income (*β* = −1813.384, p-value = 0.038) at the 1 and 5% levels, respectively. The results suggest that agricultural training decreased farm wealth and income for SSUC farmers in the ETH Municipality. Although the results may be counterintuitive, they are explainable. It could imply that farmers who received training may initially incur costs from investments in CSA practices that reduce their immediate wealth, or be in a transitional phase where they have not yet realised benefits. Regarding nutrition, this finding suggests that short-term income reductions stemming from training temporarily constrain households’ access to diverse and nutritious foods, particularly during transition or lean seasons. Similarly, it could suggest that the agricultural training received might not have been specifically relevant to implementing CSA practices that effectively lead to income growth, or that there are barriers to applying the training (101). Another plausible explanation for the negative association between agricultural training and farm income could be selection bias, in which training and extension programmes target, for example, resource-constrained, risk-prone, or low-productivity farmers, leading the CMP model to predict underlying farmer disadvantages rather than an income-training association.

The principal economic activity is negative and statistically significant at 5% level for farm income (*β* = −10,400, *p*-value = 0.025). The results suggest that SSUC farmers whose principal economic activity is formal employment experience substantially lower farm income. Farmers who are formally employed have divided attention between their formal employment and farming, leading to lower productivity on their farms and, consequently, reduced farm income. Kehinde and Ogundeji (102) report a negative correlation between farm income and non-farm employment among cassava (a DT CSA practice) farming in Nigeria. Lower farm income among formally employed SSUC farmers may limit the availability of own-produced food and dietary diversity when UA directly contributes to food consumption.

Climate-smart agricultural practices are generally labour-intensive. Hired labour negatively and statistically significantly influenced the wealth index (*β* = 0.827, *p*-value = 0.000) and farm income (*β* = −21,000, *p*-value = 0.000) at a 1% level. The negative relationship suggests that hiring labourers may impose high financial costs on SSUC farmers, reducing their wealth and farm income. Lower farm income associated with the use of hired labour presents difficulties for SSUC farmers’ ability to produce, purchase, and consume adequate, diverse, and nutritious foods, potentially undermining household nutrition outcomes. A key factor is inefficiencies in the allocation of hired labour, which can reduce productivity. Hired labour may not perform as efficiently as family labour under poor management and supervision, resulting in decreased farm output and profitability (103). High labour costs can erode profit margins, particularly in smaller-scale urban farms where labour expenses account for a significant share of total costs (104). Apart from the immediate costs of hired labour, its negative association with farm income may also be due to broader structural constraints concerning the scalability of CSA within urban contexts. This finding suggests that when CSA implementation relies on hired labour, the costs may outweigh productivity gains, which may not be feasible by SSUC farmers.

The number of part-time labourers from the household had a negative, statistically significant influence on the wealth index (*β* = −0.619, *p*-value = 0.000) and farm income (*β* = −21,200, *p*-value = 0.000) at the 1% level. The findings suggest a reduced labour force from the household available to support SSUC farmers’ farming operations. Household members working part-time may lack the time or skills to be as effective as full-time or professional labourers, hindering the accumulation of farm wealth and income (105). Lu et al. (106) noted that part-time engagement in non-farm work can dilute labour resources, leading to decreased productivity and profitability on the farm. Limited household labour availability may reduce self-production of own food, thereby undermining dietary diversity and household food and nutrition security.

The results in Table 3 show that household size had a positive and statistically significant influence on the wealth index (*β* = 0.608, *p*-value = 0.000) and farm income (*β* = 21824.010, *p*-value = 0.000) at a 1% level. The findings suggest SSUC farmers with larger households will likely be more economically productive in their UA farming operations. The current study’s findings align with those of Setsoafia et al. (77), who observed that larger farming households in Ghana with greater labour availability could adopt multiple sustainable agricultural practices, such as improved seeds and fertilisers, resulting in increased farm productivity and higher income levels. Large household sizes have the potential to improve household food and nutrition security by providing labour for food production and contributing to household income, thereby improving access to diverse foods.

Farming experience increases the likelihood of CSA adoption (first stage adoption equation of the CMP model) and boosts farm income and wealth. This finding suggests that more experienced farmers are more likely to adopt and benefit from CSA practices. Specifically, farming experience had a positive, statistically significant influence on the wealth index (*β* = 0.020, *p*-value = 0.000) and farm income (*β* = 286.260, *p*-value = 0.000) at the 1% level of significance. Haruna et al. (107) highlight that farming experience helps farmers adopt advanced agricultural technologies, leading to higher yields and greater farm income. Moreover, experienced farmers also tend to have better access to markets and credit, enabling them to make more profitable investments in their farming operations (108). Improved farm income and productivity, associated with greater farming experience, can enhance household food availability and dietary diversity by sustaining food production and ensuring access to nutritious foods.

The distance to the farming site had a positive, statistically significant effect on the wealth index (*β* = 0.092, *p*-value = 0.000) at the 1% level. The findings suggest that wealthier SSUC farmers may own or farm on more distant land, possibly due to better access to transportation or more efficient farming practices. The positive association between distance to the farming site and the wealth index might initially seem counterintuitive, as longer distances travelled by farmers could pose challenges. This finding suggests that wealthier farmers, who own or have access to larger and more distant plots of land that may be more fertile or have higher agricultural potential, are not deterred from adopting CSA practices. Wealthier farmers can afford the transportation costs or have better infrastructure to manage distant farms efficiently (109). Higher wealth levels, associated with distant farming sites, suggest more productive land, albeit at a distance, which may support improved household food production, availability, and dietary diversity.

The monthly food expenditure had a positive, statistically significant influence on the wealth index (*β* = 0.000, *p*-value = 0.000) and farm income (*β* = 0.753, *p*-value = 0.000) at the 1% significance level. Higher monthly food expenditure is associated with better access to food and higher dietary quality, which can enhance nutritional status. Beckford et al. (110) observed that wealthier households not only have more disposable income but also tend to invest in better nutrition, which, in turn, could contribute to higher productivity and well-being. In wealthier farming households, food expenditure can serve as an indicator of food security. Rashid et al. (111) confirm that higher household food expenditure, facilitated by more stable income, directly corresponds with lower levels of food insecurity in their study conducted in Tanzania.

Off-farm income had a statistically significant negative influence on the wealth index (*β* = −0.563, *p*-value = 0.000) and farm income (*β* = −10,200, *p*-value = 0.000) at the 1% level of significance. The negative association between off-farm income and the wealth index, as well as between farm income and the wealth index, suggests that SSUC farmers who rely on off-farm income may not accumulate as much wealth as those who focus solely on farming. This scenario indicates that off-farm employment may divert time and resources away from agricultural activities and investment in CSA practices, ultimately affecting overall farm output and income (112). Therefore, low farm income and weak investment in agricultural production, associated with reliance on off-farm income, suggest limited access to own-produced foods, which restrict household dietary diversity and food and nutrition security.

Employment status had a positive, statistically significant influence on the wealth index (*β* = 1.914, *p*-value = 0.000) and farm income (*β* = 46,500, *p*-value = 0.000) at the 1% level of significance. The findings suggest that SSUC farmers who are formally employed can accumulate wealth and generate more income from farming. Employment provides farmers with financial stability, enabling their households to invest in wealth-building activities such as acquiring assets, improving their homes, or saving for the future (113). Additionally, employment can significantly reduce reliance on farming alone as a source of income, allowing farmers to make more strategic, long-term investments without the pressure to achieve immediate financial returns. Samuel et al. (93), in their study examining the economic characteristics of farmers in drought-prone regions of India, found that farmers engaged in CSA practices could maintain a stable agricultural income while reducing the need to diversify into non-farm income sources. Therefore, stable income from SSUC farmers’ formal employment can enable consistent food purchases and support more diverse, nutritionally adequate diets supplemented by own-food production.

Membership in agricultural-related groups had a positive, statistically significant influence on farm income (*β* = 2392.026, *p*-value = 0.000) at the 10% level. The results imply that SSUC farmers who participate in such groups tend to earn more income from their farming activities compared to those who are not members. Setsoafia et al. (77) and Lei and Yang (114) affirm that membership in agricultural groups can significantly benefit farmers by providing access to training, shared knowledge, and best practices, thereby enhancing productivity and income. Higher farm income associated with group membership can improve household food security and dietary diversity through access to nutrition-related information, inputs, and resources that support diverse and nutritious food production and consumption.

## 5 Conclusion and recommendations

This paper assessed the contribution of CSA practices to farm income and the wealth index by SSUC farmers in the ETH Municipality. The paper used a CMP framework to jointly model CSA practices adoption and economic outcomes while accounting for selection bias. While the results may support that CSA adoption is both statistically and economically beneficial for SSUC farmers (statistically significant and positively associated with farmers’ economic welfare), the CMP model cautions that CSA adoption alone may not provide substantial short-term income benefits, may contribute incrementally to SSUC farmers’ livelihoods and longer-term economic and climatic resilience. Nonetheless, the contribution of CSA practices adoption relative to other determinants in the model highlights that CSA practices were a significant predictor of improved economic outcomes for SSUC farmers. Other crucial determinants included education, household size, farming experience, and employment status, which were significant and positively associated with both farm income and wealth. Membership in an agricultural-related group was positively associated with farm income only. Agricultural training, hired labour, part-time household labour, and off-farm income were significant but negatively associated with farm income and wealth outcomes. Access to irrigation infrastructure also exhibited a negative association with wealth outcome. These findings suggest that merely having access to, for example, irrigation infrastructure, hired labour, and agricultural training will not guarantee economic gains without effective implementation. Broadly, the findings underscore that CSA practices significantly and positively contribute to income generation and wealth outcomes by SSUC farmers. From a nutrition point of view, the findings imply that CSA adoption, when effectively implemented and accounting for socio-economic and institutional factors, can enhance household food and nutrition security by stabilising income, supporting own food production, and improving access to diverse and nutritious foods by SSUC farmers. However, farmers’ socio-economic and institutional arrangements mediate the income and wealth benefits, underscoring the need for effective CSA uptake and implementation. Based on the empirical findings, the paper advances the following recommendations:

- Efforts to promote CSA adoption by SSUC farmers are imperative. The government should redirect policy interventions toward low-cost CSA practices, such as CD, CR, M, and DT crops. Climate-smart agriculture promotion strategies should integrate nutrition objectives, embedded in low-cost CSA practices such as CD, CR, M, and DT crops, to align with the production of diverse, nutrient-dense foods and strengthen household food and nutrition security.
- There is a need to invest in farmer education aligned with CSA implementation to ensure farmers’ capacity to understand, adapt to, and optimise CSA practices to realise economic gains. This farmer educational approach is critical from a nutrition perspective, as improved knowledge and decision-making can support both income generation and the production and consumption of nutritionally diverse foods. In this case, the extension should emphasise practical CSA decision-making, farm management, food and nutrition education and CSA optimisation rather than generic farm training.
- The negative association between hired labour and farm income highlights a critical scalability challenge for CSA in urban contexts, as its economic benefits diminish when expansion relies on paid labour. Therefore, policy efforts aimed at promoting urban CSA should focus on labour-saving technologies, improved work organisation, and targeted attention. For example, this could be programmes designed to promote efficient labour use, labour-saving CSA techniques, and improved on-farm productivity, which may enhance the returns to CSA adoption. These interventions also have nutritional relevance, as improved labour efficiency can help maintain household food production and income, which are critical to supporting adequate and stable diets.
- While agricultural group membership only shows a positive association with farm income, its effect on wealth (asset accumulation) was statistically insignificant. Therefore, extension efforts could strengthen a group-based approach to CSA by focusing on economically productive functions, for example, joint marketing or input acquisition. Strengthening economically productive functions is critical to enhance nutrition outcomes by ensuring access to affordable inputs, markets, and nutrition-sensitive knowledge that support diversified food production.
- The negative association between agricultural training and farm income could result from selection bias rather than reflect the true impact of training. Support for agricultural training and access to irrigation infrastructure should be carefully redesigned and evaluated. Redesigning agricultural training presents an opportunity to align agricultural support programmes with nutrition-sensitive CSA outcomes, ensuring that productivity gains translate into improved household food and nutrition security. Therefore, policymakers should shift from assuming direct economic returns from such interventions to adopting adaptive, context-specific approaches supported by monitoring and evaluation.

Overall, these recommendations underscore that improving farm income and wealth for SSUC farmers is not merely about increasing access to CSA-related resources. Instead, contextually appropriate and efficient CSA adoption and implementation, supported by complementary socio-economic and institutional arrangements, are paramount.

## 6 Limitations of the study

This study has several limitations when interpreting the findings. The analysis relies on a quantitative cross-sectional design using data collected between 29 May and 26 June 2023, which captures CSA adoption and farm income at a single point in time; therefore, the results indicate associations rather than definitive causal effects and may not reflect seasonal or longer-term income dynamics. Although the study used a multi-stage approach, respondents were purposively selected and supplemented through snowball sampling across four wards in the ETH Municipality, which may introduce selection bias and limit the generalisability of the findings to all small-scale urban crop farmers beyond the sampled wards. While the Cochran’s formula, a probability-based sample size technique, was used to guide the minimum ideal sample size, the final sample, resulting from non-probability sampling, constitutes an inherent methodological limitation. Therefore, the findings should not be generalised as population-representative estimates, but treated as internally valid associations. Key measures, including CSA adoption practices, income, expenditure, and some institutional variables, were collected through a semi-structured questionnaire, which made the study susceptible to recall and reporting bias. Finally, while the CMP model was employed to address endogeneity and interdependence in adoption decisions, the study remains constrained by the absence of panel data and the challenges of fully accounting for unobserved factors that may jointly influence both CSA adoption and farm income outcomes. While the paper employs a CMP framework and specifies the instrumental variables to address endogeneity in CSA adoption, the validity of this approach depends on the strength and exogeneity of the exclusion restrictions. As with most cross-sectional studies, researchers cannot formally test the instruments for exogeneity beyond their statistical relevance in the first-stage adoption equation. Therefore, the risk of residual endogeneity if the instruments are correlated with unobserved determinants of income or wealth remains, calling for interpreting the CSA effects as conditional associations due to selection concerns in adoption decisions, which may not account for untested exclusion restrictions.

## Data Availability

The raw data supporting the conclusions of this article will be made available by the authors, without undue reservation.
