# Supplementary material for: The association between climate-smart agriculture practices adoption and farm income and wealth of small-scale urban crop farmers in eThekwini municipality, with implications for food and nutrition security
Source: Front Nutr. 2026 May 21;13:1792895. doi: 10.3389/fnut.2026.1792895 (PMC13235438; doi:10.3389/fnut.2026.1792895)
Supplement: Supplementary file 1 [file Supplementary_file_1.pdf]

**Appendix 1**

**PCA component loadings and summary statistics**

PCA Variables

- Access to agricultural machinery
- Access to fertiliser inputs
- Capacity to purchase produce handling materials
- Use of agricultural production technologies
- Ownership and security of farm tools and equipment
- Ownership of durable household assets

**Table 1: PCA Results for Wealth Index Construction**

| **Component** | **Eigenvalue** | **Proportion** | **Cumulative** |
|---|---|---|---|
| PC1 | 3.263 | 0.544 | 0.544 |
| PC2 | 0.936 | 0.156 | 0.700 |
| PC3 | 0.901 | 0.150 | 0.850 |
| PC4 | 0.596 | 0.099 | 0.950 |
| PC5 | 0.211 | 0.035 | 0.985 |
| PC6 | 0.092 | 0.015 | 1.000 |

Note: Following the Kaiser criterion (eigenvalue > 1), the first principal component (PC1) was retained as the wealth index.

Table 2: PCA Factor Loadings (Eigenvectors)

| **Variable** | **Comp1** | **Comp2** | **Comp3** | **Comp4** | **Comp5** | **Comp6** |
|---|---|---|---|---|---|---|
| Agricultural machinery access | 0.496 | 0.137 | 0.046 | -0.244 | -0.821 | -0.002 |
| Fertiliser input access | 0.393 | 0.025 | 0.115 | 0.900 | -0.020 | 0.149 |
| Produce handling material access | 0.504 | 0.183 | -0.008 | -0.322 | 0.429 | 0.652 |
| Agricultural technology use | 0.523 | 0.150 | 0.018 | -0.104 | 0.374 | -0.744 |
| Farm tools and equipment ownership | -0.196 | 0.484 | 0.852 | -0.036 | 0.021 | -0.005 |
| Durable household asset ownership | 0.184 | -0.831 | 0.509 | -0.123 | 0.038 | 0.011 |

Table 3: PCA Suitability Tests

| Test | Statistic |
|---|---|
| Determinant of Correlation Matrix | 0.032 |

| | |
|---|---|
| Bartlett's Test of Sphericity (Chi-square)<br><br>Degrees of Freedom<br><br>p-value | 3164.663<br><br>91<br><br>0.000 |
| Kaiser–Meyer–Olkin (KMO) | 0.905 |

Note: Comp 1 with an eigenvalue greater than 1; a cumulative of 0.544 was predicted and used as the wealth index in the CMP model. The suitability of PCA for extracting the wealth index was also assessed. The KMO and Bartlett test of sphericity results (0.9 and 0.000, respectively) indicate that PCA was suitable for extracting the wealth index, which was subsequently used as an outcome variable in the CMP model.

## Appendix 2

### Instrument validity and endogeneity diagnostics

### Falsification test

| **Distance from the water access point** | **Coef.** | **Std. Err.** | **p-value** |
|---|---|---|---|
| CSA adoption | 0.886 | 0.1992 | 0.000*** |
| Wealth index | -1.936 | 1.450 | 0.230 |
| Average income | -14183.736 | 33393.631 | 0.786 |
| Controls | Yes | | |

***** p<0.001**
